# Collagen Peptide Upregulates Osteoblastogenesis from Bone Marrow Mesenchymal Stem Cells through MAPK- Runx2

**DOI:** 10.3390/cells8050446

**Published:** 2019-05-11

**Authors:** Jeevithan Elango, Jeyashakila Robinson, Jingyi Zhang, Bin Bao, Nan Ma, José Eduardo Maté Sánchez de Val, Wenhui Wu

**Affiliations:** 1 Department of Marine Bio-Pharmacology, College of Food Science and Technology, Shanghai Ocean University, Shanghai 201306, China; srijeevithan@gmai.com (J.E.); zhangjyj@163.com (J.Z.); bbao@shou.edu.cn (B.B.); 2 Department of Fish Quality Assurance and Management, Fish Quality Monitoring and Certification Centre, Fisheries College and Research Institute, Tamil Nadu Dr.J.Jayalalitha Fisheries University, Tuticorin 628008, India; jeyashkila@gmail.com; 3 Institute of Biomaterial Science and Berlin-Brandenburg Center for Regenerative Therapies, Helmholtz-Zentrum Geesthacht, 14513 Teltow, Germany; nma@163.com; 4 Institute of Chemistry and Biochemistry, Free university Berlin, 14195 Berlin, Germany; 5 Department of Biomaterials Engineering, Universidad Católica San Antonio de Murcia, 30107 Murcia, Spain; jemate@ucam.edu; 6 Laboratory of Quality and Safety Risk Assessment for Aquatic Products on Storage and Preservation, Ministry of Agriculture, Shanghai 201306, China

**Keywords:** collagen peptide, Mahi mahi, mesenchymal stem cell, differentiation, p38MAPK, mineral deposition, Runx2

## Abstract

Collagen is the most abundant extracellular fibrous protein that has been widely used for biomedical applications due to its excellent biochemical and biocompatibility features. It is believed that the smaller molecular weight collagen, i.e., collagen peptide (CP), has more potent activity than native collagen. However, the preparation of CP from fish bone collagen is a complex and time-consuming process. Additionally, the osteogenic effect of CP depends on its molecular weight and amino acid composition. Considering the above concept, the present work was undertaken to extract the CP directly from Mahi mahi fish (*Coryphaena hippurus*) bones and test its osteogenic potential using bone marrow mesenchymal stem (BMMS) cells. The hydrolyzed collagen contained triple alpha chains (110 kDa) and a peptide (~1 kDa) and the peptide was successfully separated from hydrolyzed collagen using molecular weight cut-off membrane. CP treatment was up-regulated BMMS cells proliferation and differentiation. Interestingly, CP accrued the mineral deposition in differentiated BMMS cells. Protein and mRNA expression revealed that the osteogenic biomarkers such as collagen, alkaline phosphatase, and osteocalcin levels were significantly increased by CP treatment in differentiated BMMS cells and also further elucidated the hypothesis that CP was upregulated osteogenesis through activating Runx2 via p38MAPK signaling pathway. The above results concluded that the CP from Mahi mahi bones with excellent osteogenic properties could be the suitable biomaterial for bone therapeutic application.

## 1. Introduction

There are three major types of protein present in fish, such as sarcoplasmic (25–30%), myofibrillar (66–77%) and stroma protein (1–5%). Among them, stromal proteins are mostly located in the interstitial space of muscle cells and extra-cellular membrane. Collagen is one of the most available stromal proteins in fish. Collagen can be extracted from different part of fish such as skin, scales, bones, muscles, fins, and swim bladder [1,2,3].

In recent years, collagens from marine organism have been widely investigated due to their excellent biological activity with low or no side effects as an alternative to mammalian collagen [4,5]. The quality of collagen or gelatin (partially denatured form of collagen) depends on its biochemical, functional, and rheological properties, which may differ depending on fish species and manufacturing method [6]. Based on the quality and bioactivity, the fish collagen may be used as a food supplement for special medical purposes [7,8].

To explore more regarding fish collagen, many researchers are focusing on the biological activity of collagen peptides at present. For instance, Chen et al. [9] investigated the arthritogenicity and tolerogenicity effect of collagen peptide with 12 kDa. Xi et al. [10] reported that oral administration of a novel recombinant peptide rcCTE1-2 (50 μg/kg/d) with 28 kDa induced immune tolerance and suppressed rheumatoid arthritis (RA). Earlier, Schmid and Conrad [11] identified a new collagen peptide (59 kDa) that involved in calcification of the cartilage. It has been demonstrated that collagen peptides possess the ability to suppress T-cell activation in RA both in vitro and in vivo [12].

Recently, few studies have been focused on the preparation and biomedical application of type-II collagen polypeptides from cartilaginous fish species [13,14,15]. A study revealed that collagen chicken sternal cartilage collagen peptides (0.5–3 kDa) had anti-oxidant, anti-inflammatory activity and inhibited chondrocytes apoptosis [16]. Enteral nutritional administration of Alaska pollock skin collagen peptides ranging from ca. 0.7–1 kDa showed protective effects on intestinal tight junction after burn injury in mice model [17]. It is believed that the antioxidant and the anti-inflammatory response of peptides are regulated by the specific amino acid sequences [6,8].

It has been reported that certain specific modulators such as mitogen-activated kinases (MAPKs) regulate skeletal development and bone homeostasis, and play main role in osteoblast differentiation from bone marrow-mesenchymal stem (BMMS) cells [18,19,20]. There are three types of MAPKs, such as the c-Jun amino (N)-terminal kinases (JNK1/2/3), extracellular signal-regulated kinases (ERK1/2, ERK5), and the p38 isoforms (p38α, p38β, p38γ, and p38δ) [21]. Among three MAPKs, the role of p38 and ERK in skeleton development is predominantly investigated [22,23,24,25].

In most of the previous studies, the collagen peptides were obtained by proteolytic digestion of fish collagen extracted from fish parts [9,13,14,15]. However, for the first time, we have initiated the extraction of collagen peptide directly from fish bone through proteolytic digestion. To the best of our knowledge, the osteogenic properties of Mahi mahi bone collagen peptide have not yet been reported. Therefore, the present study was aimed to extract collagen peptides directly from Mahi mahi fish bones through various pretreatments and alcalase hydrolysis, and test the osteogenic behavior of collagen peptide using mouse BMMS cells.

## 2. Results

### 2.1. Molecular Weight Analysis

**Figure 1:**
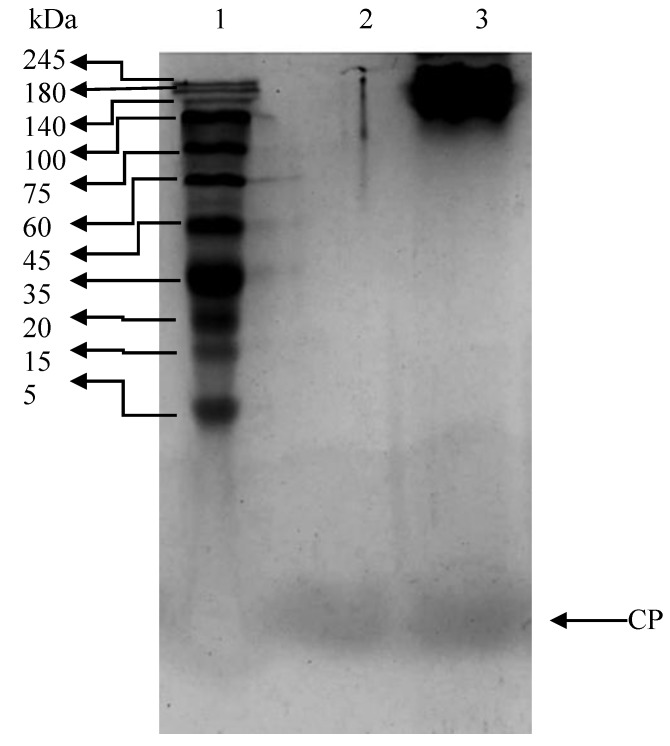
Tricine SDS-PAGE pattern of Mahi mahi bone collagen. Lane 1: protein standard marker; lane 2: collagen peptide; lane 3: collagen hydrolysate.

The electrophoretic patterns of Mahi mahi bone collagen hydrolysate and peptide are shown in Figure 1. Collagen hydrolysate had polypeptide chain with the molecular weight of ~150 kDa and a peptide. On the basis of the electrophoretic mobilities, the α-chain of the bone collagen hydrolyzed by alcalase was degraded into smaller peptide with a molecular weight of ~1 KDa. From the electrophoretic pattern, it was clearly seen that the hydrolysis of bone by alcalase could efficiently produce a peptide with the molecular weight of ~1 kDa.

### 2.2. Effect of CP on the Proliferation of BMMS Cells

**Figure 2:**
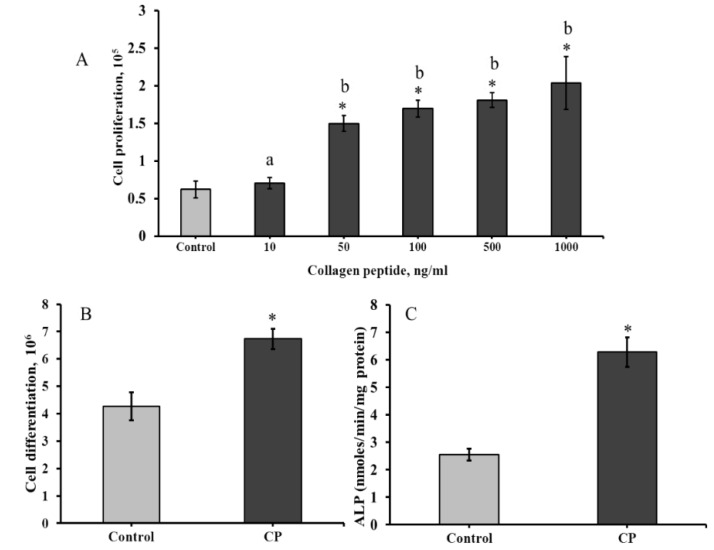
Effect of collagen peptide on proliferation (**A**), differentiation (**B**), and cellular alkaline phosphatase level (**C**) of bone marrow mesenchymal stem cells. CP-collagen peptide. * *p* < 0.05 vs. control.

The effect of CP on mouse bone marrow-mesenchymal stem (BMMS) cells growth was determined and presented in Figure 2. As shown in Figure 2A, the CP treated BMMS cells showed high cell proliferative rate than control cells, except 10 ng/mL CP treated group. The proliferation rate of BMMS cells was significantly increased with increasing concentration of CP in dose dependent manner. Notably, the proliferative rate was significantly higher at 50 ng/mL of CP, however, there was no significant difference between 50 ng/mL and other higher doses of CP (100–1000 ng/mL).

### 2.3. Effect of CP on the Differentiation of BMMS Cells

BMMS cells were differentiated in osteoblast growth medium (osteoblastogenic medium and supplement) for 21 days. In order to investigate the osteogenic differentiation ability, BMMS cells were treated with CP (50 ng/mL) in presence of osteoblast growth medium. The differentiation of BMMS cells was up-regulated 1.8 fold by CP compared to the control group (*p* < 0.05) (Figure 2B). For further confirmation, the cellular level of alkaline phosphatase (ALP), a hallmark biomarker for osteoblast differentiation, was analyzed in BMMS cells after the CP treatment. As expected, the cellular ALP level was significantly up-regulated in CP treated BMMS cells than control cells (Figure 2C), which further substantiate the osteogenic differentiation ability of CP.

### 2.4. Histological Staining

**Figure 3:**
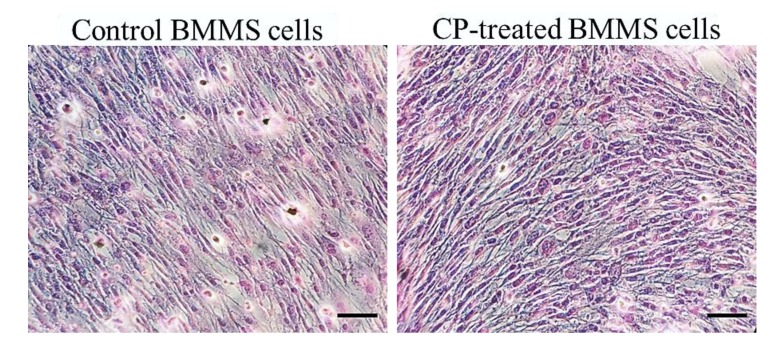
Haematoxylin and eosin staining of control and collagen peptide (CP)-treated bone marrow mesenchymal stem (BMMS) cells. Scale bars: 100 micrometers.

**Figure 4:**
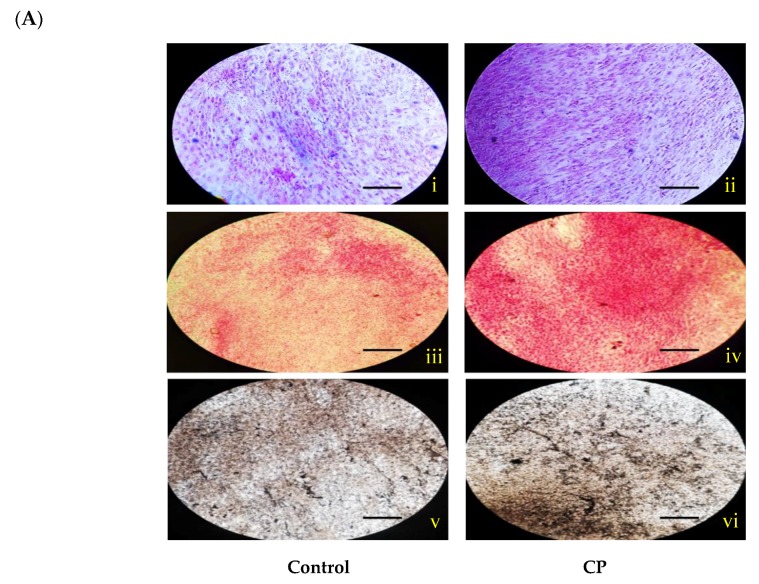

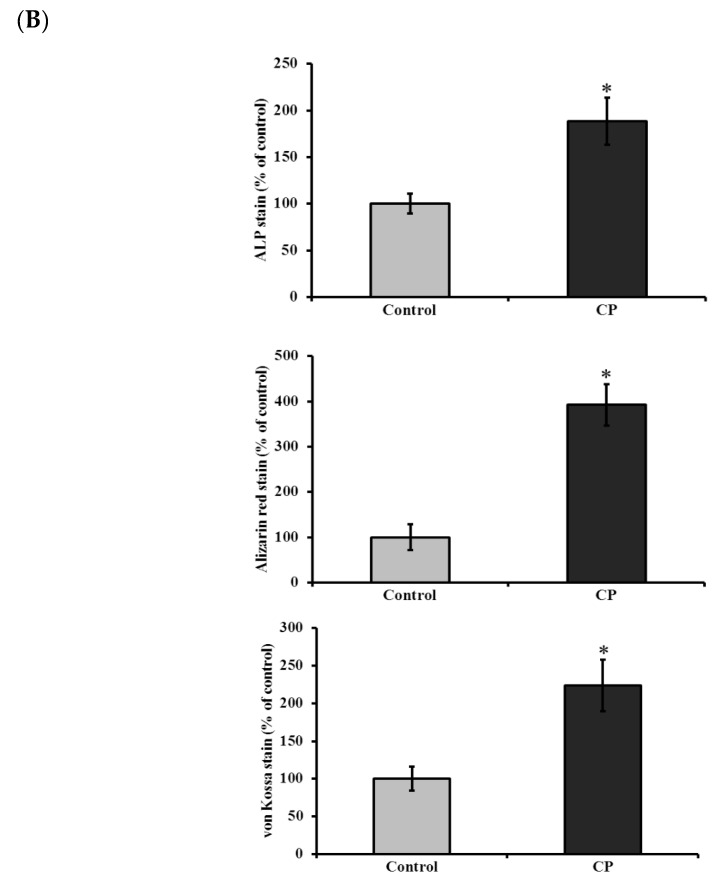
(**A**) Histological staining for alkaline phosphatase (i–ii), alizarin red (iii–vi) and von Kossa (v–vi) of control and collagen peptide (CP)-treated bone marrow mesenchymal stem cells (scale bars: 0.1 cm). (**B**) Quantification of stained area of bone marrow mesenchymal stem cells. The percentage of stained area in bone cells was quantified using ImageJ software (Version 1.52n). CP-collagen peptide, * *p* < 0.05 vs. control.

Histological staining (H and E stain) of CP treated and control BMMS cells are shown in Figure 3. It was clearly shown that the number of BMMS cells was increased in CP treated cells than control cells. Histological staining of naphthol AS-MX phosphate-fast blue RR for alkaline phosphatase showed that on day 21, the CP treated BMMS cells had high deposition of ALP compared to control cells (*p* < 0.05) (Figure 4).

In addition, histological mineral staining of BMMS cells using alizarin red and von Kossa stain (silver nitrate) showed the existence of high level of nodular red and apatite black precipitate in the extracellular matrix of CP treated BMMS cells than control cells on day 21 (*p* < 0.05), however, there were no significant changes observed between CP-treated BMMS cells and control cells on day 7 and 14 (Supplementary Figures S1 and S2).

### 2.5. Immunocytochemistry

To examine the effect of CP on the expression of an osteogenic protein in BMMS cells, we used immunocytochemistry with antibodies directed against osteogenic protein such as Col_1_α_2_.

**Figure 5:**
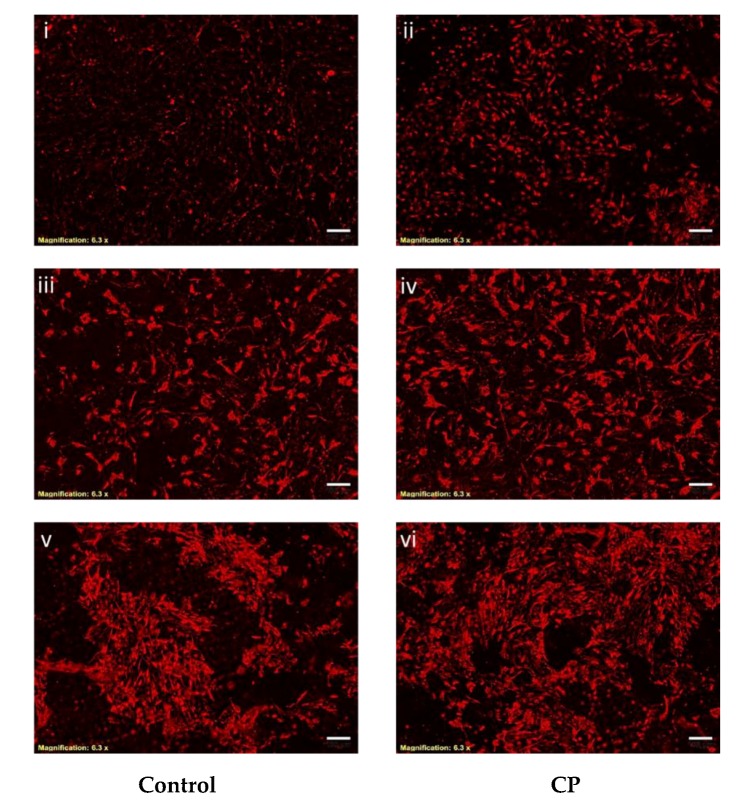
Immunocytochemistry of control and collagen peptide (CP)-treated bone marrow mesenchymal stem cells. Bone marrow mesenchymal stem cells treated with primary antibody (anti-Col_1_α_2_) overnight and DyLight 594-conjugated secondary antibody (scale bars: 75 micrometers). I–ii, iii–iv, and v–vi: 7, 14 and 21 days treated BMMS cells, respectively.

This approach demonstrated that Col_1_α_2_ was increased in CP treated BMMS cells compared to control cells. In general, the expression of collagen was significantly increased in 21 days cultured control BMMS cells compared to seven and 14 days of culture. However, BMMS cells cultured with CP showed strong staining with Col_1_α_2_ monoclonal antibody than control BMMS cells after 21 days of culture (Figure 5), which also supported the osteogenic differentiation of BMMS cells cultured with CP.

### 2.6. mRNA and Protein Expression of CP Treated BMMS Cells

**Figure 6:**
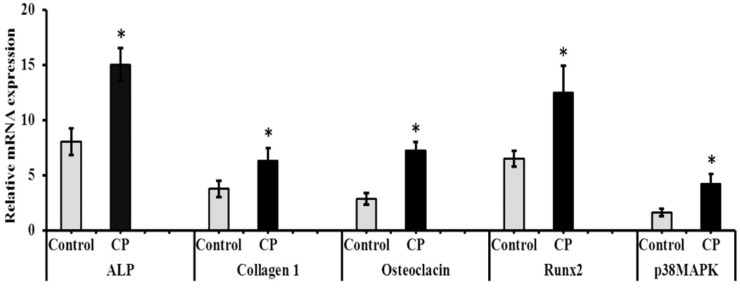
Osteogenic mRNA expression of collagen peptide-treated bone marrow mesenchymal stem cells. ALP: alkaline phosphatase; CP: collagen peptide. * *p* < 0.05 vs. control.

**Figure 7:**
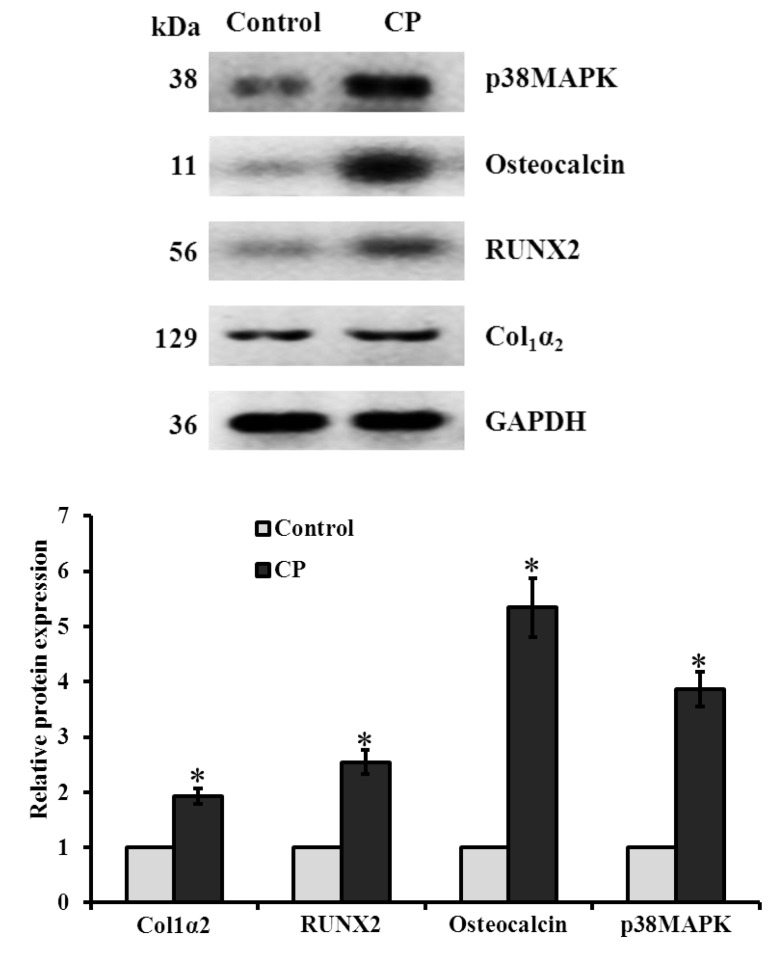
Western blot analysis of collagen peptide (CP)-treated BMMS cells. * *p* < 0.05 vs. control.

To determine the mRNA expression, BMMS cells cultured with CP in presence of osteogenic medium for 21 days and the genes of interest measured by RT-PCR were normalized with a house-keeping gene, GAPDH. The level of osteogenic regulatory mRNA (Col_1_α_2_, ALP and osteocalcin (OC)) and protein (Col_1_α_2_ and osteocalcin) expression was significantly increased in CP treated cells on day 21 compared to control BMMS cells (Figure 6 and Figure 7). To further investigate the mechanism leading to the differentiation of osteogenic cells by CP, the levels of osteogenic signaling modulators, such as Runx2 and p38MAPK were measured. Our results confirmed that Runx2 and p38MAPK levels were significantly increased in BMMS cells cultured with CP compared to control cells (*p* < 0.05) (Figure 7), which further disclose the possible mechanism of BMMS cells differentiation by CP.

## 3. Materials and Methods

### 3.1. Extraction of Fish Bone Collagen Peptide (CP)

**Scheme 1:**
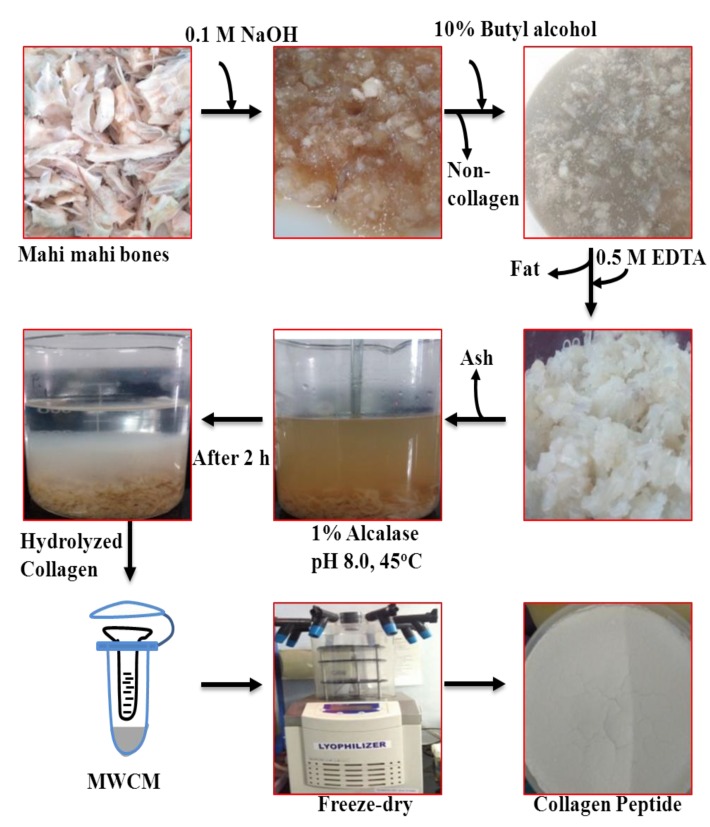
Schematic illustration of extraction steps involved in isolation of collagen peptide from Mahi mahi bone. MWCM: molecular weight cut-off membrane.

The schematic representation of collagen peptide extraction is shown in Scheme 1. In brief, Fish (Mahi mahi, *Coryphaena hippurus*) bones were treated with 0.1 M NaOH for 24 h to remove non-collagenous protein. After, they were treated with 10% Butyl alcohol for 24 h to remove fat molecules and then treated with 0.5 M EDTA to remove ash content. The bones were washed thoroughly in tap-water between each step. The pretreated bones were incubated with 1% alcalase (Sigma-Aldrich, Shanghai, China) in water with the ratio of 1:2 and were hydrolyzed at 45 °C, pH 8.0 for 2 h. The hydrolyzed collagen samples were collected and collagen peptides were separated using 10 KDa molecular weight cut-off membrane (MWCM) (Millipore, Shanghai, China) by ultra-centrifugation. The fractions were freeze dried using a lyophilizer and stored in air tight container for further use.

### 3.2. Molecular Mass by Tricine SDS-PAGE

Tricine-SDS-PAGE was done according to a method described by Schagger [26]. Briefly, the freeze dried collagen hydrolysate and its fraction were mixed with the sample buffer (1M Tris-HCl, pH 6.8, containing 20% SDS, glycerol and Coomassie Brilliant Blue), heated at 90 °C for 15 min and was loaded (10 μL) onto a polyacrylamide gel, composed of 5% of stacking and 15% of separating gels and were subjected to electrophoresis at constant current of 100 mA. Protein standard (5–245 kDa) was also run on the gels. After electrophoresis, the gels were incubated with staining solution (Coomassie Blue, methanol and acetic acid) and de-staining solution (acetic acid).

### 3.3. Cell Culture

Mouse bone marrow-mesenchymal stem (BMMS) cells were obtained from the Shanghai Zhong Qiao Xin Zhou Biotechnology Co., Ltd., Shanghai, China and were cultured in mesenchymal stem cell culture medium (cat. no. 7501) containing 10% fetal bovine serum (FBS) (cat. no. 0025), mesenchymal stem cell growth supplement (1% MSCGS, cat. no. 7552) and 1% penicillin/streptomycin (10,000 units/mL of penicillin and 10,000 μg/mL of streptomycin in a saline solution) (cat. no. 0503) at 37 °C in a CO_2_ incubator.

### 3.4. Effect of CP on BMMS Cells Proliferation

BMMS cells (5000 cells/well) were seeded in Corning^®^ TC-Treated 48 well plates and were treated with different concentration of collagen peptide (10, 50, 100, 500 and 1000 ng/mL) for seven days. Control cells were grown in the mesenchymal stem cell culture medium without a sample. The total number of viable cells was counted using an Invitrogen cell counter (Countess II Automated Cell Counter, ThermoFisher Scientific, Shanghai, China).

### 3.5. Effect of CP on BMMS Cells Differentiation

BMMS cells at passage 5 were initially seeded at a density of 5 × 10^4^ cells/well in microtiter six-well plates. To induce osteogenesis, the cells were grown in osteoblast medium (Shanghai Zhong Qiao Xin Zhou Biotechnology Co., Ltd., cat. no. 4601) with the addition of osteoblast growth supplement (ObGS) (Shanghai Zhong Qiao Xin Zhou Biotechnology Co., Ltd., Shanghai, China, cat. no. 4652) composed of 100 nM dexamethasone, 10 mM b-glycerolphosphate, and 0.05 mM 2-phosphate-ascorbic acid for 21 days with media changes every three days. To evaluate the CP osteogenic property, BMMS cells were cultured in osteogenic medium with collagen peptide (50 ng/mL). After 7, 14, and 21 days of culture, the cells were counted and fixed with respective fixatives as mention below.

### 3.6. Histological Staining for Osteogenic Evaluation

At the end of CP treatment, the cells were washed with DPBS (consist without calcium and magnesium) to remove non-adherent cells and fixed with 0.4% paraformaldehyde (PFA) for 30 min, after that the cell membranes were permiabilized by 0.1% Triton-X 100 in PBS at room temperature for 30 min. In each step, the excessive fixative was washed out using DPBS. The fixed cells were then stained with various staining agents such as hematoxylin and eosin, naphthol AS-MX phosphate and fast blue RR for alkaline phosphatase and alizarin red and silver nitrate stain for mineral deposition [27,28].

### 3.7. Cellular Alkaline Phosphatase

The level of cellular alkaline phosphatase (ALP) was measured as per the previous protocol [29]. The cells were harvested with lysis buffer (10 mM Tris buffer, pH 7.4) and treated with ALP substrate, p-nitrophenyl phosphate (Sigma-Aldrich) and read at 410 nm using a plate reader (Bio-Rad Model 550, Shanghai, China). The same volume of sample was used to determine protein content using bicinchoninic acid (BCA) as per the manufacturer’s instruction (Pierce, Missouri, IL, USA). ALP activity was expressed as nmoles/min/mg protein.

### 3.8. Immunocytochemistry

The cells were grown in the confocal disc (cat no. 150682, ThermoFisher Scientific) with CP for 7, 14, and 21 days and fixed as mentioned earlier. Then, the cells were incubated with primary antibody (anti-Col_1_α_2_) overnight and DyLight 594-conjugated secondary antibody (goat anti-rabbit IgG H&L, Cat No. ab96885, Abcam) for 2 h at 37 °C. Images were captured using confocal laser scanning microscope (Leica TCS SP8, Leica Microsystems CMS GmbH, Wetzlar, Germany).

### 3.9. mRNA Expression

BMMS cells (1 × 10^5^ cells/well) were cultured in six-well plates with CP as mentioned above. The percentage of mRNA expression (ALP, osteocalcin, Runx2, p38MAPK, and GAPDH) was determined as follows.

#### 3.9.1. RNA Extractions

Culture medium was removed from the cells by pipetting after an incubation period and washed twice with ice-cold PBS. The cell monolayer was lysed in TRIzol (Invitrogen Life Technologies, Shanghai, China) (0.5 mL/well). The Trizol supernatant was passed through a 21 G needle using Luer-lock syringes (Zogear Industries Co., Ltd., Shanghai, China) to shear the RNA. Phase separation was obtained by addition of 0.2 mL chloroform for each 1 mL of Trizol used and shaken vigorously by hand until the sample was opaque. Then cells were left on ice for a few min and spun at 12,000× *g* for 15 min at 4 °C. The top clear phase containing RNA was collected and added to a fresh tube containing 0.5 mL isopropanol for RNA precipitation. The mixture was mixed thoroughly and centrifuged as previously. The pellet was washed with 0.5 mL 70% ethanol and re-centrifuged. The RNA pellet was suspended in an appropriate volume (10 μL) of RT-PCR water (Shanghai Biocolor BioScience and Technology Company, Shanghai, China). DNase treatment was done using a Turbo DNase kit (Shanghai Biocolor BioScience and Technology Company, Shanghai, China) as per the manufacturer’s instructions. After centrifuging at 1000× *g* for 2 min at 4 °C, RNA was quantified at an absorbance of 260 nm using a Nanodrop ND-1000 spectrophotometer (ThermoFisher Scientific).

#### 3.9.2. cDNA Synthesis and RT-PCR

First strand cDNA synthesis was done in a 20 μL reaction mixture containing 1.0 μg RNA, 10 μL FS master mix (Invitrogen, Shanghai, China), 3 μL random primer (Invitrogen), 1 μL affinity script (Invitrogen) and molecular-grade water to 20 μL. The reaction mixture was incubated at 42 °C for 30 min and 85 °C for 5 min, followed by 5 min at 90 °C to inactivate the RT in a Biometra T3000 thermocycler 48 (Biometra Gmbh, Gottingen, Germany).

Real-time polymerase chain reaction (RT-PCR) was done using a 96-well plate using an ABI 7500 Fast Real-Time PCR System (Applied Biosystems, Shanghai, China) using SYBR Green Fast qPCR RT Master Mix (Invitrogen, Shanghai, China). The total volume of each PCR reaction was 10 μL, containing 5 μL SYBR Green Fast qPCR RT Master mix, 1.5 μL cDNA template sample, 0.5 μL of forward and reverse primers, and 2.5 μL water. The PCR reaction was carried out at 95 °C for 30 min, 40 cycles at 95 °C for 5 min, 60 °C for 30 min, one cycle of 95 °C for 1 h, 55 °C for 30 min and 95 °C for 30 min. The primers used for the RT-PCR are given below.

GAPDH: forward 5′- AGC TTG TCA TCA ACG GGA AG-3′ and reverse 5′- TTT GAT GTT AGT GGG GTC TCG-3′

COL I: forward 5′- GCG AAG GCA ACA GTC GCT-3′ and reverse 5′- CTT GGT GGT TTT GTA TTC GAT GAC-3′

Osteocalcin (OC): forward 5′- CTC ACA GAT GCC AAG CCC-3′ and reverse 5′- CCA AGG TAG CGC CGG AGT CT-3′

Alkaline Phosphatase (ALP): forward 5′- TCC TGA CCA AAA ACC TCA AAG G-3′ and reverse 5′- TGC TTC ATG CAG AGC CTG C-3′

RUNX2: forward 5′- CCA CCA CTC ACT ACC ACA CG-3′ and reverse 5′- TCA GCG TCA ACA CCA TCA TT-3′

p38MAPK: forward 5′-TCGAGACCGTTTCAGTCCAT-3′ and reverse 5′-CCA CGG ACC AAA TATCCA CT-3′.

### 3.10. Western Blot

BMMS cells (1 × 10^5^ cells/well) cultured in six-well plates were treated with CP as mentioned earlier. At the end of treatment, total cell proteins were extracted using RIPA buffer (strong) containing Protease inhibitor cocktail (Roche-Complete™, Mini, EDTA-free Protease Inhibitor Cocktail Tablets) (Sigma, Shanghai, China). The cell lysates were used to measure the total protein content using BCA method. The protein fragments were separated 10% SDS-PAGE and transferred to PVDF nitrocellulose membranes (Invitrogen) using the iblot-2 dry blotting system (Invitrogen). 5% BSA-PBST was used as blocking agent before antibodies treatment. Then, the PVDF membranes were incubated with primary antibodies (Abcam, Shanghai, China) such as anti-GAPDH (ab245355), anti-RUNX2 (ab76956), anti-p38MAPK (ab27986), anti-Col_1_α_2_ (ab96723), and anti-osteocalcin (ab93876) overnight at 4 °C. Polyclonal goat anti-rabbit IgG-HRP (ab6721) as secondary antibody incubated with membrane for 2 h at 37 °C. The bands were visualized by exposing with the enhanced chemiluminescent reagent using a Universal Hood II Gel Doc System (Bio-Rad, Rochester, NY, USA).

### 3.11. Statistics

The average mean values and standard deviation of the mean were calculated from three determinations and statistical significance was determined using one-way ANOVA. Individual differences between mean values were assessed using Duncan’s multiple range tests. The results were also statistically interpreted using SPSS 18.0 (SPSS 18.0 for Windows, SPSS Inc, Chicago, IL, USA) to determine the least significant differences (LSD) at a *p* < 0.05. Each experiment was repeated thrice.

## 4. Discussion

Though the proteolytic enzyme alcalase could effectively hydrolyze the bone collagen, the alpha chain of collagen was still intact, which means the part of high molecular weight component was hydrolyzed by alcalase and degraded into smaller peptide with the molecular weight of 1 kDa. Similar to the present study, collagen peptides with the molecular weight of 0.5–3 kDa were isolated from chicken sternal cartilage through papain enzymatic hydrolysis and tested its antioxidant, anti-inflammatory, and chondrogenic properties [16]. In another study, the protective effects of Alaska pollock skin collagen peptides (0.7–1 kDa MW) on intestinal tight junction were investigated through diminished intestinal barrier disruption after burn injury [17]. In the present study, the electrophoretic pattern disclosed that the alcalase enzyme could effectively hydrolyze the bone collagen and produce the single major peptide with a molecular weight of ~1 kDa.

Interestingly, the proliferation and differentiation rate of BMMS cells were increased by CP treatment and the effect of CP on BMMS cells was dose-dependent, however, the optimum concentration of CP for BMMS cells proliferation was 50 ng/mL since there was no significant difference in BMMS cells treated with CP from 50–1000 ng/mL. Similar to the present study, collagen peptides (1.4, 2, and 5 kDa) from bovine, porcine, and fish had upregulated the proliferation and ALP activities of human osteoblastic MG-63 and MC3T3-E1 cells [30,31,32]. It was reported that type I collagen mimetic peptides coated hydroxyapatite disks and rat tail type I collagen showed osteogenic stimulatory activities on mesenchymal stem cells [33,34,35]. Recently, Chiu et al. [36] reported that type-II collagen treatment increased the level of integrin α_2_β_1_ complex (VLA-2) expression in BMMS cells surface.

CP accelerated the cellular ALP and mineral deposition in BMMS cells compared to other controls, which confirmed the osteoblastogenic potential of this material. Similarly, mammalian collagen-treated BMMS cells accrued more calcium than control in the previous study [36]. Daneault et al. [37] reported that hydrolyzed collagen rich in hydroxyproline had increased the mineralization of osteoblasts in vitro.

Immunocytochemistry data using Col_1_α_2_ monoclonal antibody confirmed that the BMMS cells treated with CP were increased cellular collagen level than control. It has been opined that the higher expression of collagen supported calcification of stromal cell matrix in bone [38,39]. In our study, we have also found higher levels of collagen and mineral deposition in differentiated BMMS cells on day 21, which was further supported through higher expression of collagen, ALP and osteocalcin in RT-PCR and Western blot analysis. These results may be explained by the fact that the higher level of collagen in differentiated CP-treated BMMS cells may accelerate the deposition of minerals. Similarly, an earlier report claimed that tripeptides (Gly-Pro-Hyp) of porcine skin collagen had upregulated the mineral, collagen, and osterix expression in human osteoblast hFOB1.19 cells [40]. In another study, type I collagen treatment had upregulated the osteoblast differentiation regulatory gene expression in bone marrow cells during the osteoblastic differentiation [41,42,43].

An earlier in vivo study revealed that salmon fish collagen peptides (100–860 Da) supplementation promoted the development of long bones in growing male rats through higher level of mineral, osteocalcin and ALP of osteoblasts [44]. Several in vitro studies also reported that collagen peptides from porcine skin (3 and 5 kDa), fish (1 kDa), chicken foot (800 Da), and bovine (2 and 5 kDa) had upregulated the bone growth and limited the bone damages during normal and arthritis conditions [30,31,32,37,45,46,47,48].

To find the exact signaling mechanism of BMMS cells differentiation by CP, the level of osteoblast differentiation transcription factor, Runx2 and p38MAPK expression was determined in CP-treated BMMS cells. Culturing BMMS cell with CP increased Runx2 and p38MAPK expression in BMMS cells, which answered the possible scenario behind the osteogenic stimulatory mechanism of CP. From these observations, we opined that the differentiation of BMMS cells by CP achieved by activation of Runx2 via p38MAPK signaling pathway.

It was earlier confirmed by few authors that certain specific amino acid domain (asparagine, glycine, glutamine, and alanine) of collagen interact with α_2_β_1_ integrin on the bone marrow mesenchymal stem cell membrane and can lead to up-regulate bone matrix synthesis through activation of Runx2 signaling pathway [29,36,41,49,50]. Kawaguchi et al. [51] observed the presence of radioactive dipeptides [14C] Pro-Hyp in osteoblasts, osteoclasts, dermal fibroblasts, epidermal cells, synovial cells, and chondrocytes after 24 h of oral administration. More specifically, collagen-derived dipeptides containing Pro-Hyp had promoted osteoblastic MC3T3-E1 cells differentiation through upregulation of osteogenic Foxg1 expression [52].

Hence, this study summaries that, the CP obtained by direct extraction from Mahi mahi bone upregulated the BMMS cells proliferation as well as osteogenic differentiation, which is the main advantages of this CP. Though the present study disclosed the role of Mahi mahi bone CP on osteogenic differentiation of BMMS cells in vitro, it is necessary to understand the molecular mechanism of this CP with other bone cells such as osteoblasts, osteocytes, chondrocytes, and osteoclasts, which is one of the limitations of this study. The other limitation of this study is lack of in vivo evidence to prove the osteogenic effect of this CP, which needs further extensive research.

## 5. Conclusion

In the present study, a new methodology was adopted to obtain bone collagen hydrolysates directly from fish bone through pretreatments with 0.1 M NaOH, 10% butyl alcohol and 0.5 M EDTA and proteolytic enzyme hydrolysis by 1% alcalase, rather than extracting the collagen from fish bone and then obtaining the peptide through the hydrolysis of collagen. From this new method, we obtained a specific peptide with the molecular weight of ~1 kDa. Most importantly, the obtained CP promoted osteoblast cells growth and upregulated collagen and mineral synthesis in bone cells, which is a most desirable property of biomaterials for the treatment of the bone disorder. We further confirmed that CP triggers p38MAPK dependent runx2 signaling pathway in BMMS cells during osteoblast differentiation. Overall, the fish CP with good osteogenic stimulatory properties may be considered as a potential drug for the osteoporotic treatment in old age and menopause women. However, one important issue that needs to be addressed by further studies is the angiogenesis effect of this CP using an in vivo study in order to understand the mechanism of action in bone regeneration.

## Supplementary Materials

The Supplementary Materials are available online at https://www.mdpi.com/2073-4409/8/5/446/s1.

## References

[1] Liu D., Liang L., Regenstein J.M., Zhou P. (2012). Extraction and characterisation of pepsin-solubilised collagen from fins, scales, skins, bones and swim bladders of bighead carp (*Hypophthalmichthys nobilis*). Food Chem..

[2] Nagai T., Suzuki N. (2000). Isolation of collagen from fish waste material - skin, bone and fins. Food Chem..

[3] Muralidharan N., Jeya Shakila R., Sukumar D., Jeyasekaran G. (2013). Skin, bone and muscle collagen extraction from the trash fish, leather jacket (*Odonus niger*) and their characterization. J. Food Sci. Technol..

[4] Jeevithan E., Bao B., Bu Y., Zhou Y., Zhao Q., Wu W.H. (2014). Type II collagen and gelatin from silvertip shark (*Carcharhinus albimarginatus*) cartilage: Isolation purification physicochemical and antioxidant properties. Mar. Drug.

[5] Jongjareonrak A., Benjakul S., Visessanguan W., Nagai T., Tanaka M. (2005). Isolation and characterisation of acid and pepsin-solubilised collagens from the skin of brownstripe red snapper (*Lutjanus vitta*). Food Chem..

[6] Gómez-Guillén M.C., Giménez B., López-Caballero M.E., Montero M.P. (2011). Functional and bioactive properties of collagen and gelatin from alternative sources: A review. Food Hydrocoll..

[7] Jeevithan E., Zhao Q., Bin B., Wu W. (2013). Biomedical and pharmaceutical application of fish collagen and gelatin: A Review. J. Nutr. Therapeut..

[8] Liu D., Nikoo M., Boran G., Zhou P., Regenstein J.M. (2015). Collagen and gelatin. Ann. Rev. Food Sci. Technol..

[9] Chen L., Bao B., Wang N., Xie J., Wu W.H. (2012). Oral administration of shark type II collagen suppresses complete freund’s adjuvant-induced rheumatoid arthritis in rats. Pharmaceutical.

[10] Xi C., Tan L., Sun Y., Liang F., Liu N., Xue H., Luo Y., Yuan F., Sun Y., Xi Y. (2009). A novel recombinant peptide containing only two T-cell tolerance epitopes of chicken type-II collagen that suppresses collagen-induced arthritis. Mol. Immunol..

[11] Schmid T.M., Conrad H.E. (1982). A unique low molecular weight collagen secreted by cultured chick embryo chondrocytes. J. Biol. Chem..

[12] Myers L.K., Sakurai Y., Rosloniec E.F., Stuart J.M., Kang A.H. (2004). An analog peptide that suppresses collagen-induced arthritis. Am. J. Med. Sci..

[13] Ehrlich H. (2015). Cartilage of marine vertebrates. Biol. Mater Marine Orig..

[14] Xie J., Ye H.Y., Luo X.F. (2014). An efficient preparation of chondroitin sulfate and collagen peptides from shark cartilage. Int. Food Res. J..

[15] Jeevithan E., Bao B., Zhang J., Hong S., Wu W. (2015). Purification, characterization and antioxidant properties of low molecular weight collagenous polypeptide (37 kDa) prepared from whale shark cartilage (Rhincodon typus). J. Food Sci. Technol..

[16] Wang J., Luo D., Liang M., Zhang T., Yin X., Zhang Y., Yang X., Liu W. (2018). Spectrum-effect relationships between high-performance liquid chromatography (HPLC) fingerprints and the antioxidant and anti-inflammatory activities of collagen peptides. Molecule.

[17] Chen Q., Gao X., Zhang H., Li B., Yu G., Li B. (2019). Collagen peptides administration in early enteral nutrition intervention attenuates burn-induced intestinal barrier disruption: Effects on tight junction structure. J. Funct. Food..

[18] Rodríguez-Carballo E., Gámez B., Ventura F. (2016). p38 MAPK signaling in osteoblast differentiation. Front Cell Dev. Biol..

[19] Xu X., Han J., Ito Y., Bringas P., Deng C., Chai Y. (2008). Ectodermal Smad4 and p38 MAPK are functionally redundant in mediating TGF-beta/BMP signaling during tooth and palate development. Dev. Cell.

[20] Pizzicannella J., Diomede F., Merciaro I., Caputi S., Tartaro A., Guarnieri S., Trubiani O. (2018). Endothelial committed oral stem cells as modelling in the relationship between periodontal and cardiovascular disease. J. Cell Physiol..

[21] Cargnello M., Roux P.P. (2011). Activation and function of the MAPKs and their substrates, the MAPK-activated protein kinases. Microbiol. Mol. Biol. Rev..

[22] Sciandra M., Marino M.T., Manara M.C., Guerzoni C., Grano M., Oranger A., Lucarelli E., Lollini P.-L., Dozza B., Pratelli L. (2014). CD99 drives terminal differentiation of osteosarcoma cells by acting as a spatial regulator of ERK 1/2. J. Bone Miner Res..

[23] Xiao G., Jiang D., Gopalakrishnan R., Franceschi R.T. (2002). Fibroblast growth factor 2 induction of the osteocalcin gene requires MAPK activity and phosphorylation of the osteoblast transcription factor, Cbfa1/Runx2. J. Biol. Chem..

[24] Cuadrado A., Nebreda A.R. (2010). Mechanisms and functions of p38 MAPK signalling. Biochem. J..

[25] Brancho D., Tanaka N., Jaeschke A., Ventura J.J., Kelkar N., Tanaka Y., Kyuuma M., Takeshita T., Flavell R.A., Davis R.J. (2003). Mechanism of p38 MAP kinase activation in vivo. Genes Dev..

[26] Schagger H. (2006). Tricine-SDS-PAGE. Nature.

[27] Donzelli E., Salvadè A., Mimo P., Viganò M., Morrone M., Papagna R., Carini F., Zaopo A., Miloso M., Baldoni M. (2007). Mesenchymal stem cells cultured on a collagen scaffold: In vitro osteogenic differentiation. Arch Oral. Biol..

[28] Zhang Z., Yin S., Xue X., Ji J., Tong J., Goltzman D., Miao D. (2016). Transplantation of bone marrow-derived mesenchymal stem cells rescues partially rachitic phenotypes induced by 1, 25-Dihydroxyvitamin D deficiency in mice. Am. J. Transl. Res..

[29] Jeevithan E., Sanchez C., de Val J.M.E.S., Henrotin Y., Wang S., Motaung K.S.C.M., Regenstein J.M., Bao B., Wu W.H. (2018). Cross-talk between primary osteocytes and bone marrow macrophages for osteoclastogenesis upon collagen treatment. Sci. Rep..

[30] Kim H.K., Kim M.G., Leem K.H. (2013). Osteogenic activity of collagen peptide via ERK/MAPK pathway mediated boosting of collagen synthesis and its therapeutic efficacy in osteoporotic bone by back-scattered electron imaging and microarchitecture analysis. Molecule.

[31] Guillerminet F., Beaupied H., Fabien-Soule V., Tome D., Benhamou C.L., Roux C., Blais A. (2010). Hydrolyzed collagen improves bone metabolism and biomechanical parameters in ovariectomized mice: An in vitro and in vivo study. Bone.

[32] Daneault A., Coxam V., Fabien Soulé V., Wittrant Y. Hydrolyzed collagen contributes to osteoblast differentiation in vitro and subsequent bone health in vivo. Presented at the OARSI World Congress.

[33] Fathi-Najafi M., Vahedi F., Ahmadi S., Madani R., Mehrvarz M. (2008). Effect of collagen type I (Rat tail) on cell proliferation and adhesion of BHK-21. 4th Kuala Lumpur. Int. Conf. Biomed. Eng..

[34] Gao C., Harvey E.J., Chua M., Chen B.P., Jiang F., Liu Y., Li A., Wang H., Henderson J.E. (2013). MSC-seeded dense collagen scaffolds with a bolus dose of VEGF promote healing of large bone defects. Euro. Cells Mat..

[35] Hennessy K.M., Pollot B.E., Clem W.C., Phipps M.C., Sawyer A.A., Culpepper K., Bellis S.L. (2009). The effect of collagen I mimetic peptides on mesenchymal stem cell adhesion and differentiation, and on bone formation at hydroxyapatite surfaces. Biomaterial.

[36] Chiu L.H., Lai W.F., Chang S.F., Wong C.C., Fan C.Y., Fang C.L. (2014). The effect of type II collagen on MSC osteogenic differentiation and bone defect repair. Biomaterial.

[37] Daneault A., Prawitt J., Fabien Soulé V., Coxam V., Wittrant Y. (2017). Biological effect of hydrolyzed collagen on bone metabolism. Crit. Rev. Food Sci. Nutr..

[38] Bernhardt A., Lode A., Boxberger S., Pompe W., Gelinsky M. (2008). Mineralised collagen-An artificial, extracellular bone matrix—Improves osteogenic differentiation of bone marrow stromal cells. J. M. Sci. Mat. Med..

[39] Tsuruoka N., Yamato R., Sakai Y., Yoshitake Y., Yonekura H. (2007). Promotion by collagen tripeptide of type I collagen gene expression in human osteoblastic cells and fracture healing of rat femur. Biosci. Biotechnol. Biochem..

[40] Boskey A.L., Robey P.G. (2013). Chapter 11—The regulatory role of matrix proteins in mineralization of bone. Osteopores (Fourth Edition).

[41] Mizuno M., Kuboki Y. (2001). Osteoblast-related gene expression of bone marrow cells during the osteoblastic differentiation induced by type I collagen. J. Biochem..

[42] Jeevithan E., Jung W.L., Shujun W., Yves H., José E.M.S.V., Regenstein J.M., Bao B., Wu W.H. (2018). Evaluation of differentiated bone cells proliferation by blue shark skin collagen via biochemical for bone tissue engineering. Mar. Drug.

[43] Mizuno M., Fujisawa R., Kuboki Y. (2000). Type I collagen-induced osteoblastic differentiation of bone-marrow cells mediated by collagen-alpha2beta1 integrin interaction. J. Cell Physiol..

[44] Xu Y., Han X., Li Y. (2010). Effect of marine collagen peptides on long bone development in growing rats. J. Sci. Food Agric..

[45] Wu J., Fujioka M., Sugimoto K., Mu G., Ishimi Y. (2004). Assessment of effectiveness of oral administration of collagen peptide on bone metabolism in growing and mature rats. J. Bone Miner Metab..

[46] Takeda S., Park J.H., Kawashima E., Ezawa I., Omi N. (2013). Hydrolyzed collagen intake increases bone mass of growing rats trained with running exercise. J. Int. Soc. Sports Nutr..

[47] Watanabe-Kamiyama M., Shimizu M., Kamiyama S., Taguchi Y., Sone H., Morimatsu F., Komai M. (2010). Absorption and effectiveness of orally administered low molecular weight collagen hydrolysate in rats. J. Agric. Food Chem..

[48] Guillerminet F., Fabien-Soule V., Even P.C., Tome D., Benhamou C.L., Roux C., Blais A. (2012). Hydrolyzed collagen improves bone status and prevents bone loss in ovariectomized C3H/HeN mice. Osteoporos Int..

[49] Tuckwell D.S., Ayad S., Grant M.E., Takigawa M., Humphries M.J. (1994). Conformation dependence of integrin-type II collagen binding Inability of collagen peptides to support alpha 2 beta 1 binding, and mediation of adhesion to denatured collagen by a novel alpha 5 beta 1-fibronectin bridge. J. Cell Sci..

[50] Zhang Z., Zhou S., Mei Z., Zhang M. (2017). Inhibition of p38MAPK potentiates mesenchymal stem cell therapy against myocardial infarction injury in rats. Mol. Med. Rep..

[51] Kawaguchi T., Nanbu P.N., Kurokawa M. (2012). Distribution of prolylhydroxyproline and its metabolites after oral administration in rats. Biol. Pharm. Bull..

[52] Kimira Y., Odaira H., Nomura K., Taniuchi Y., Inoue N., Nakatani S., Shimizu J., Wada M., Mano H. (2017). Collagen-derived dipeptide prolylhydroxyproline promotes osteogenic differentiation through Foxg1. Cell Mol. Biol. Lett..

