# Supplementary material for: Collagen Peptide Upregulates Osteoblastogenesis from Bone Marrow Mesenchymal Stem Cells through MAPK- Runx2
Source: Cells. 2019 May 11;8(5):446. doi: 10.3390/cells8050446 (PMC6562845; doi:10.3390/cells8050446)
Supplement: Supplementary file 1 [file cells-08-00446-s001.pdf]

Collagen peptide upregulates osteoblastogenesis from bone marrow mesenchymal stem cells through MAPK- Runx2

Jeevithan Elango [1], Jeyashakila Robinson [2], Jingyi Zhang [1], Bin Bao [1], Nan Ma [3,4], José Eduardo Maté Sánchez de Val [5] and Wenhui Wu [1,6,*]

[1] Department of Marine Bio-Pharmacology, College of Food Science and Technology, Shanghai Ocean University, Shanghai 201306, China; srijeevithan@gmai.com (J.E.); zhangjyj@163.com (J.Z.); bbao@shou.edu.cn (B.B.)

[2] Department of Fish Quality Assurance and Management, Fish Quality Monitoring and Certification Centre, Fisheries College and Research Institute, Tamil Nadu Dr.J.Jayalalitha Fisheries University, Tuticorin 628008, India; jeyashkila@gmail.com

[3] Institute of Biomaterial Science and Berlin-Brandenburg Center for Regenerative Therapies, Helmholtz-Zentrum Geesthacht, Teltow 14513, Germany

[4] Institute of Chemistry and Biochemistry, Free university Berlin, Berlin 14195, Germany; nma@163.com

[5] Department of Biomaterials Engineering, Universidad Católica San Antonio de Murcia, Murcia 30107 , Spain; jemate@ucam.edu

[6] Laboratory of Quality and Safety Risk Assessment for Aquatic Products on Storage and Preservation, Ministry of Agriculture, Shanghai 201306, China

* Correspondence: whwu@shou.edu.cn; Tel.: +86-216-190-0388; Fax: +86-216-190-0364

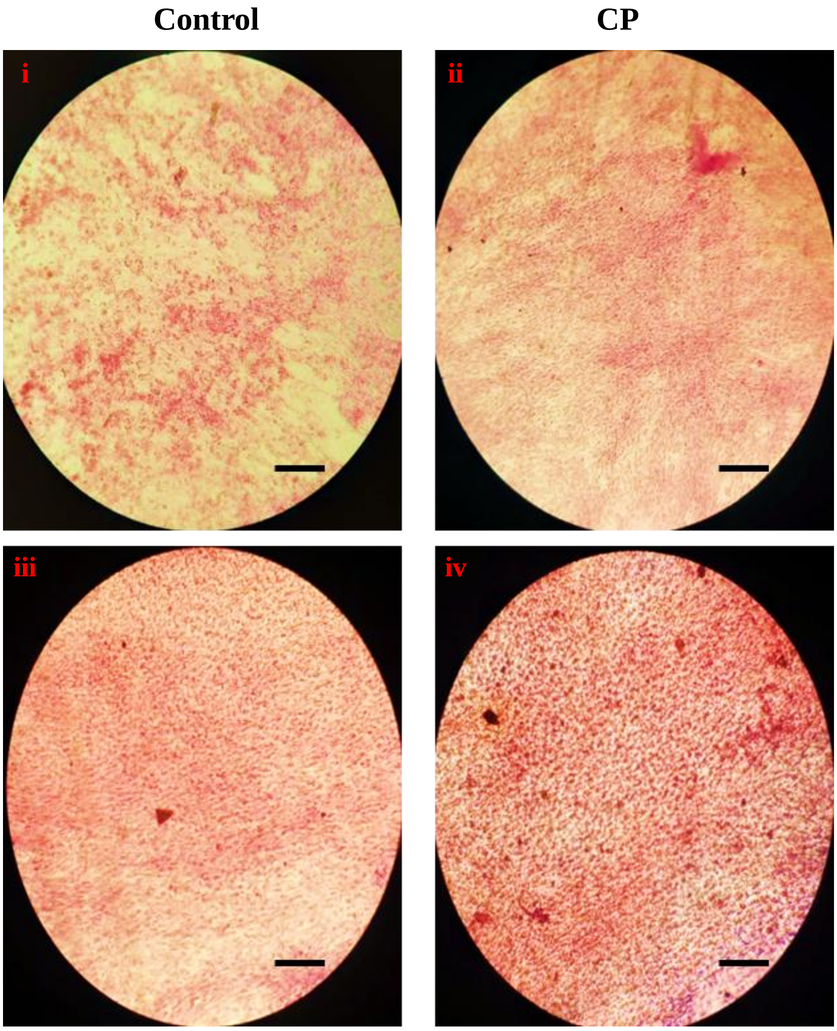

Supplementary Fig.1. Alizarin red staining of differentiated BMMS cells (i-ii: 7 days & iii-iv: 14 days treated BMMS cells), Scale bars: 0.1 cm. CP-collagen peptide.

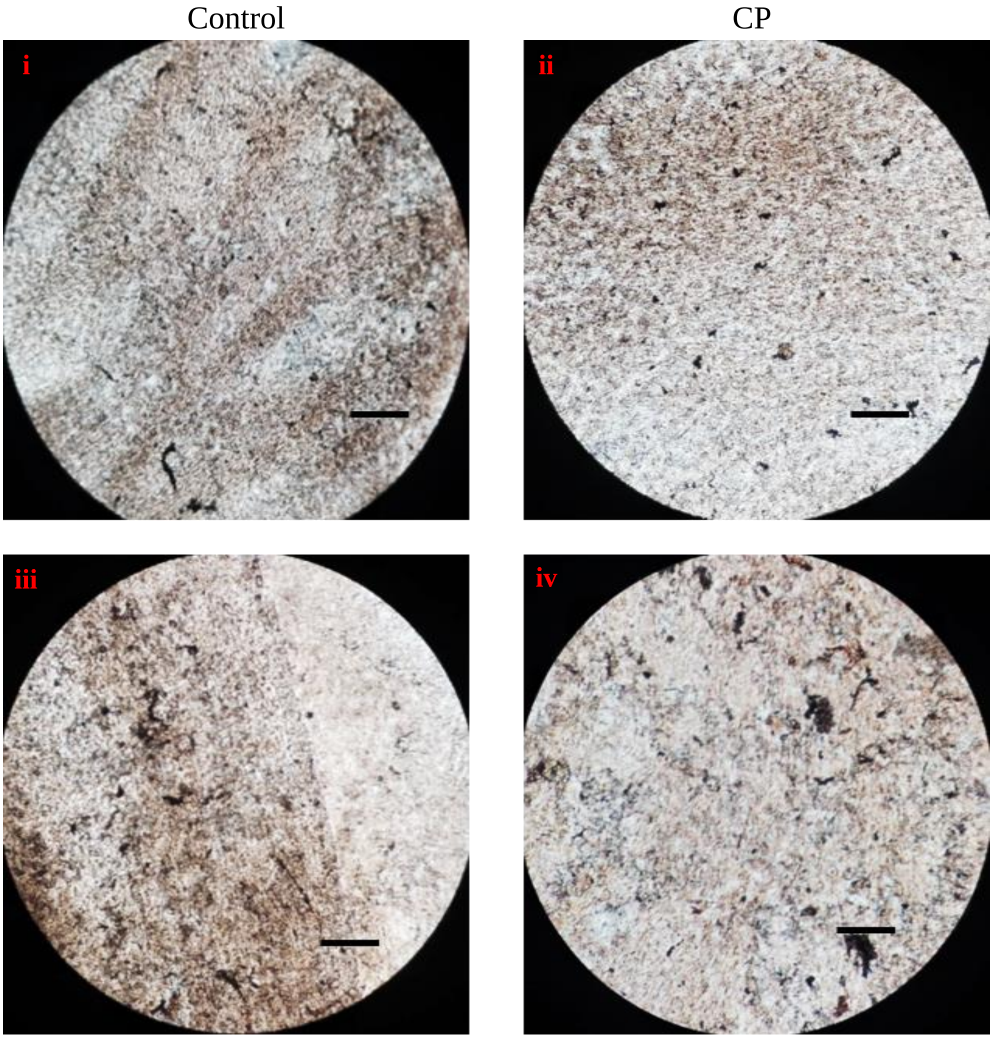

Supplementary Fig.2. von Kossa staining of differentiated BMMS cells (i-ii: 7 days & iii-iv: 14 days treated BMMS cells), Scale bars: 0.1 cm. CP-collagen peptide.
